# Brain Microstructural Alterations in Children Post‐COVID‐19 Infection Through VBM, SBM, and Structural Covariance Network Analysis

**DOI:** 10.1002/brb3.70905

**Published:** 2025-09-21

**Authors:** Rui Wang, Jing Liu, Jiayi Li, Xinmao Ma, Chuan Fu, Hui Zhang, Lekai Luo, Gang Ning, Yi Liao, Fenglin Jia, Haibo Qu

**Affiliations:** ^1^ Department of Radiology West China Second University Hospital Sichuan University Chengdu Sichuan People's Republic of China; ^2^ Key Laboratory of Birth Defects and Related Diseases of Women and Children (Sichuan University), Ministry of Education, West China Second University Hospital Sichuan University Chengdu Sichuan People's Republic of China; ^3^ Department of Oncology Incaier Sichuan Friendship Hospital Chengdu Sichuan People's Republic of China

**Keywords:** brain connectivity, children, COVID‐19, neurodevelopment, structural covariance network

## Abstract

**Background:**

Children represent a particularly vulnerable group to the long‐term consequences of COVID‐19 due to their ongoing neurodevelopment. This study aimed to identify transient and persistent structural alterations in children recovering from the infection by comparing pretreatment and posttreatment MRI scans and to evaluate differences in brain morphology and network organization relative to age‐ and sex‐matched healthy controls.

**Methods:**

A retrospective cohort of 26 children aged 8–12 years with confirmed COVID‐19 was compared to 26 healthy controls. All participants underwent high‐resolution T1‐weighted MRI on a 3T scanner using identical acquisition protocols. Standard VBM and SBM pipelines were applied to quantify cortical volume, thickness, and sulcal depth, followed by SCN analysis to construct correlation matrices based on gray matter metrics. Graph theoretic metrics, including clustering coefficients, eigenpath lengths, small‐worldness, and global/local efficiencies, were computed under different network sparsity thresholds.

**Results:**

Cortical volume analyses revealed reductions in regions including the cingulate cortex, hippocampus, and superior temporal gyrus among children post‐COVID‐19, with within‐group comparisons showing decreases in the left middle cingulate cortex (7.4–6.9 cm^3^), left postcentral gyrus (12.2–10.8 cm^3^), and right anterior cingulate cortex (2.1–1.8 cm^3^). Partial recovery of sulcal depth and cortical thickness was observed in the superior temporal gyrus (sulcal depth from 210.3 to 198.5 mm^2^, thickness from 2.34 to 2.15 mm). Structural covariance network analysis demonstrated lower global efficiency and higher small‐worldness in the post‐COVID‐19 group compared to controls, along with increased characteristic path length, whereas local connectivity measures (clustering coefficient and local efficiency) remained relatively stable.

**Conclusions:**

Children recovering from COVID‐19 may exhibit structural brain changes and network connectivity disruptions, some of which show partial resolution over time, whereas others persist. Long‐term follow‐up through comprehensive neuroimaging and clinical evaluation is necessary to clarify the potential impact on development.

## Graphical abstract

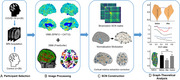

## 1 Introduction

The global COVID‐19 pandemic has had profound effects on public health, with children representing a particularly vulnerable population due to their ongoing physical and neurodevelopmental maturation. While much attention has focused on respiratory and systemic complications of the virus, there is an emerging body of evidence highlighting its neurological impact, including cognitive, emotional, and behavioral changes (Wei et al. 2023, Ariza et al. 2024, Bower et al. 2022). These concerns are particularly pronounced in children, whose brains are undergoing critical periods of development, suggesting that even transient viral effects could have long‐term consequences (Siddique et al. 2022, Khan et al. 2022).

Neuroimaging studies in adults recovering from COVID‐19 have consistently reported structural and functional brain alterations, including reductions in gray matter (GM) volume in regions such as the orbitofrontal cortex and parahippocampal gyrus, along with disruptions in connectivity within critical networks (Guo et al. 2024, Campabadal et al. 2023, Pan et al. 2023). However, the relevance of these findings to pediatric populations remains unclear due to the distinct neuroplasticity of the developing brain, which may render it simultaneously vulnerable and resilient to external insults (Schober et al. 2021, Pavel et al. 2022). Childhood is marked by dynamic processes of brain maturation, including cortical remodeling and connectivity refinement, which are sensitive to environmental and biological stressors (Casey et al. 2005). Despite preliminary evidence suggesting that children recovering from COVID‐19 may experience symptoms such as headaches, cognitive difficulties, and behavioral changes, the structural brain alterations underlying these observations remain poorly characterized (Morrow et al. 2021, Ng et al. 2022). Moreover, COVID‐19's impact on neuroinflammation and vascular integrity raises the possibility of widespread and diffuse brain alterations, which may not be captured through traditional region‐specific analyses (Neves et al. 2024).

Traditional neuroimaging methods, such as voxel‐based morphometry (VBM) and surface‐based morphometry (SBM), are well‐established and effective techniques for quantifying structural brain changes. VBM allows for the assessment of volumetric changes in gray and white matter (WM), while SBM focuses on surface‐level metrics such as cortical volume, thickness, and sulcal depth, providing complementary insights into brain morphology (Ashburner and Friston 2000, Fischl et al. 1999). Beyond these traditional methods, structural covariance network (SCN) analysis has emerged as a powerful tool for examining interregional connectivity patterns in the brain. SCNs model the degree to which brain regions exhibit coordinated variations in structural properties, reflecting shared developmental, maturational, and pathological influences (Alexander‐Bloch, Raznahan, et al. 2013, Alexander‐Bloch, Giedd, et al. 2013). This approach captures the mesoscale organization of the brain, bridging the gap between local morphometric changes and global network alterations. SCN analysis is particularly sensitive to disruptions in brain development, as it considers systemic changes rather than isolated regional effects (Aboud et al. 2019), making it highly suited for studying complex conditions such as COVID‐19‐related brain alterations. For instance, SCNs have been shown to reveal patterns of cortical reorganization and connectivity disruptions in neurodevelopmental and neurological disorders (Spreng et al. 2019, Zarkali et al. 2021). Metrics derived from SCN analysis, including global and local efficiency, small‐worldness, and nodal centrality, offer valuable insights into the functional implications of structural disruptions (Rubinov and Sporns 2010).

In this study, we aim to comprehensively characterize both transient and persistent structural brain changes following COVID‐19 infection by comparing pretreatment and posttreatment MRI data of children with COVID‐19 and contrasting these findings with those from a healthy control group. The results will advance our understanding of the impact of COVID‐19 on neurodevelopment in children and provide a foundation for future clinical monitoring and intervention strategies to mitigate potential long‐term consequences.

## 2 Methods

### 2.1 Participants

This retrospective study included 26 children who recovered from COVID‐19 infection and 26 age‐ and sex‐matched healthy controls. The experimental group consisted of children aged 8–12 years (mean = 10.5, SD = 3.65), identified from medical records between December 2020 and December 2022, who were diagnosed with COVID‐19 via reverse transcription polymerase chain reaction (RT‐PCR) according to World Health Organization protocols. All participants in the experimental group had confirmed positive results for COVID‐19 nucleic acid testing, and their clinical presentations ranged from asymptomatic to mild or ordinary cases. Fever was a common symptom, with temperatures ranging from 37.6°C to 38.5°C. Blood investigations varied, including normal results, elevated white blood cell counts, or high inflammatory markers. Among these participants, 11 underwent follow‐up MRI scans, with an average interval of approximately 4 months (mean = 125 days, range: 21–180 days) following confirmed COVID‐19 infection, to assess longitudinal changes in brain structure, while the remaining children had only one MRI scan. Participants in the experimental group were screened to exclude severe COVID‐19 encephalopathy, and baseline MRI results showed no significant abnormalities.

**FIGURE 1:**
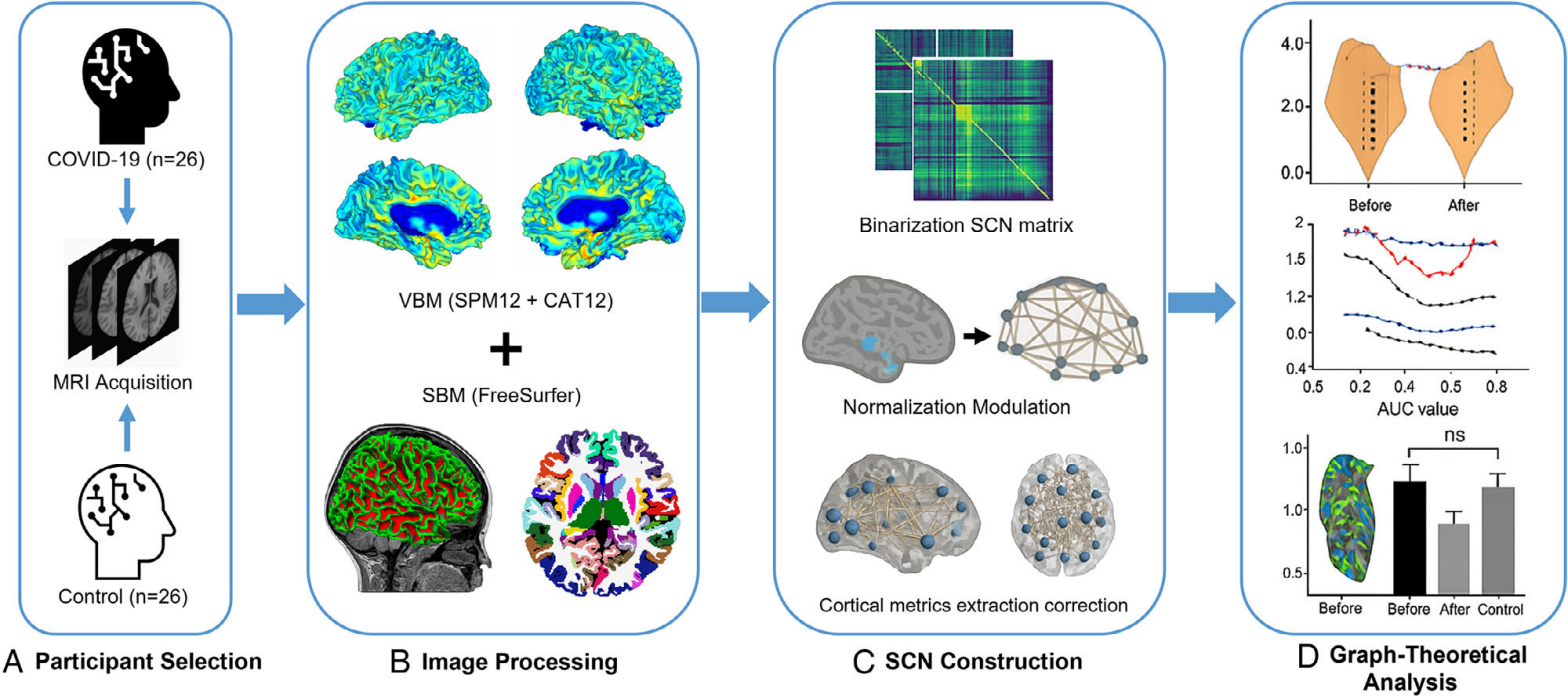
Analysis framework of this study. (A) MRI data were acquired using high‐resolution T1‐weighted imaging; (B) VBM and SBM pipelines were applied to extract cortical volume, thickness, and sulcal depth; (C) SCNs were constructed based on interregional correlations of morphometric features, followed by graph‐theoretical analysis of network topology; (D) Group metrics were compared using graph‐theoretical analysis.

The healthy control group comprised age‐ and sex‐matched children with no history of COVID‐19 infection or related symptoms, recruited through local community outreach programs (mean = 10.9, SD = 4.12). Inclusion criteria required no preexisting neurological or psychiatric disorders and no significant medical conditions. Exclusion criteria for both groups encompassed the presence of brain lesions on MRI, significant motion artifacts in imaging data, prior head trauma, congenital neurological abnormalities, or a history of neurodevelopmental or psychiatric disorders. All participants provided demographic and clinical data, including vaccination status, which indicated that most of the experimental group had been vaccinated. This study was approved by the Institutional Review Board of West China Second University Hospital (Approval No.: K2019048). Written informed consent was obtained from parents or guardians, with assent provided by the children where applicable. Table S1 summarizes the demographic and clinical characteristics of the participants. To provide an overview of the experimental workflow, the analysis framework of this study is summarized in Figure 1.

### 2.2 MRI Data Acquisition

MRI data were acquired for all participants using a 3T Siemens Skyra scanner equipped with a 32‐channel phase‐array head coil. Structural images of the brain were obtained using a high‐resolution T1‐weighted magnetization‐prepared rapid gradient‐echo (MP2RAGE) sequence with the following parameters: repetition time (TR) = 2300 ms, echo time (TE) = 2.98 ms, inversion time (TI) = 900 ms, flip angle = 9°, field of view (FOV) = 256 × 240 mm, voxel size = 1 × 1 × 1 mm, and 176 sagittal slices covering the entire brain (Marques et al. 2010). To ensure consistency, all scans were conducted using the same protocol across participants. To reduce motion artifacts, children were positioned with foam pads to secure the head and were allowed to watch age‐appropriate cartoons during scanning. Sedation was not used for any participants. All T1‐weighted images were visually inspected by three experienced imaging technicians for head motion and artifacts. Additionally, image quality was quantitatively assessed using the Computational Anatomy Toolbox (CAT12, http://dbm.neuro.uni‐jena.de/cat12/) in the Statistical Parametric Mapping (SPM12, https://www.fil.ion.ucl.ac.uk/spm/software/spm12/) framework. Homogeneity of segmented GM maps was evaluated, with maps exhibiting low homogeneity (mean < mean – 2 × SD across subjects) undergoing additional manual inspection. High‐quality images were selected for further processing, ensuring accurate representation of cortical structures.

### 2.3 Image Processing

This study utilized MATLAB (R2022a, MathWorks, Natick, MA, United States) with the CAT12 and SPM12 toolkits for VBM and SBM analyses. MRI data in DICOM format were converted to NIfTI format using the MRIcron toolkit (https://www.nitrc.org/projects/mricron) for compatibility with subsequent processing steps. For VBM, the analysis included (1) segmentation of the brain into GM, WM, and cerebrospinal fluid (CSF) using tissue probability maps (TPM) and the DARTEL algorithm; (2) spatial normalization of GM images to the Montreal Neurological Institute (MNI) standard space; (3) modulation to preserve volumetric information during normalization; and (4) smoothing of GM maps with an 8‐mm full‐width at half‐maximum (FWHM) Gaussian kernel. SBM analysis was conducted using FreeSurfer (version 7.1.0, http://surfer.nmr.mgh.harvard.edu/), including the following steps: (1) alignment and spatial normalization to MNI space; (2) segmentation of cortical and subcortical structures into GM, WM, and CSF, with corrections applied for intensity inhomogeneities and noise; (3) reconstruction of cortical surfaces to calculate cortical volume, thickness, and sulcal depth; (4) parcellation of the cerebral cortex into 68 regions based on the Desikan–Killiany atlas; and (5) smoothing of cortical thickness and sulcal depth maps using a 15‐mm smoothing kernel.

### 2.4 SCN Construction

SCNs were constructed to investigate interregional brain connectivity patterns based on GM structural metrics. Cortical and subcortical regions were parcellated into 68 cortical areas and additional subcortical regions using the Desikan–Killiany atlas in the FreeSurfer pipeline (Desikan et al. 2006). Cortical metrics, including cortical thickness, volume, and sulcal depth, were extracted and corrected for age, sex, and total intracranial volume (TIV) using linear regression models to minimize confounding effects. To construct SCNs, Pearson's correlation coefficients (𝑟) were calculated between corrected regional values across all participants for each pairwise region. The formula for the Pearson correlation coefficient is as follows:

$$r=\frac{\sum_{i=1}^{n}\left(X_{i}-\bar{X}\right)\left(Y_{i}-\bar{Y}\right)}{\sqrt{\sum_{i=1}^{n}{\left(X_{i}-\bar{X}\right)}^{2}}\sqrt{\sum_{i=1}^{n}{\left(Y_{i}-\bar{Y}\right)}^{2}}}$$

Where $X_{i}$ and $Y_{i}$ are the structural metrics of regions X and Y for participant i, $\bar{X}$, and $\bar{Y}$ are their respective mean values, and n is the total number of participants. This process resulted in correlation matrices representing the interregional covariance. Negative correlations were excluded due to their ambiguous neurobiological interpretation and inconsistent directionality, particularly in developing brains where out‐of‐phase maturation or measurement noise may result in spurious anticorrelations. This exclusion also ensured the resulting network matrices were biologically interpretable and methodologically comparable across participants. The correlation matrices were binarized and thresholded based on specific network sparsity levels, defined as the ratio of existing edges to the maximum possible edges in a network. The sparsity range for this study was set between 0.14 and 0.5 (with intervals of 0.02), ensuring that all networks remained fully connected without isolated nodes while avoiding non‐biological network configurations. Global and local graph‐theoretical parameters were computed using the thresholded SCNs. Global parameters included clustering coefficient (*Cp*), normalized Cp (*Gamma*), characteristic path length (*Lp*), normalized Lp (*Lambda*), small‐world scalar (*Sigma*), global efficiency (*Eg*), and local efficiency (*Eloc*). Local parameters, such as node degree and betweenness centrality, were also calculated. All calculations were performed using the Graph Theory Analysis Toolkit in MATLAB, with quality control steps such as visual inspection and adjustment of the adjacency matrix.

### 2.5 Statistical Analysis

All statistical analyses were conducted using MATLAB and associated toolkits, including the Brain Connectivity Toolbox and in‐house scripts tailored for SCN analysis. Group‐level comparisons were performed between children post‐COVID‐19 infection and age‐ and sex‐matched healthy controls across cortical metrics and SCN measures. Statistical significance was defined at *p *< 0.05, corrected for multiple comparisons using the false discovery rate (FDR) method where applicable. To evaluate differences in cortical volume, thickness, and sulcal depth, multivariable linear regression models were employed with covariates for age, sex, and TIV. For SCN measures, network robustness, and global and local graph‐theoretical properties, permutation testing with 5000 iterations was used to evaluate between‐group differences. Permutation tests involved shuffling group labels to generate null distributions for each network metric. Observed differences were compared against the permutation distribution to compute two‐tailed *p* values. The impact of node removal on network integrity was quantified by measuring changes in the size of the largest connected component, with area under the curve (AUC) analyses used to summarize results. Statistical results were visualized using BrainNet Viewer and other MATLAB‐based tools.

## 3 Results

### 3.1 Cortical Volume Changes

**FIGURE 2:**
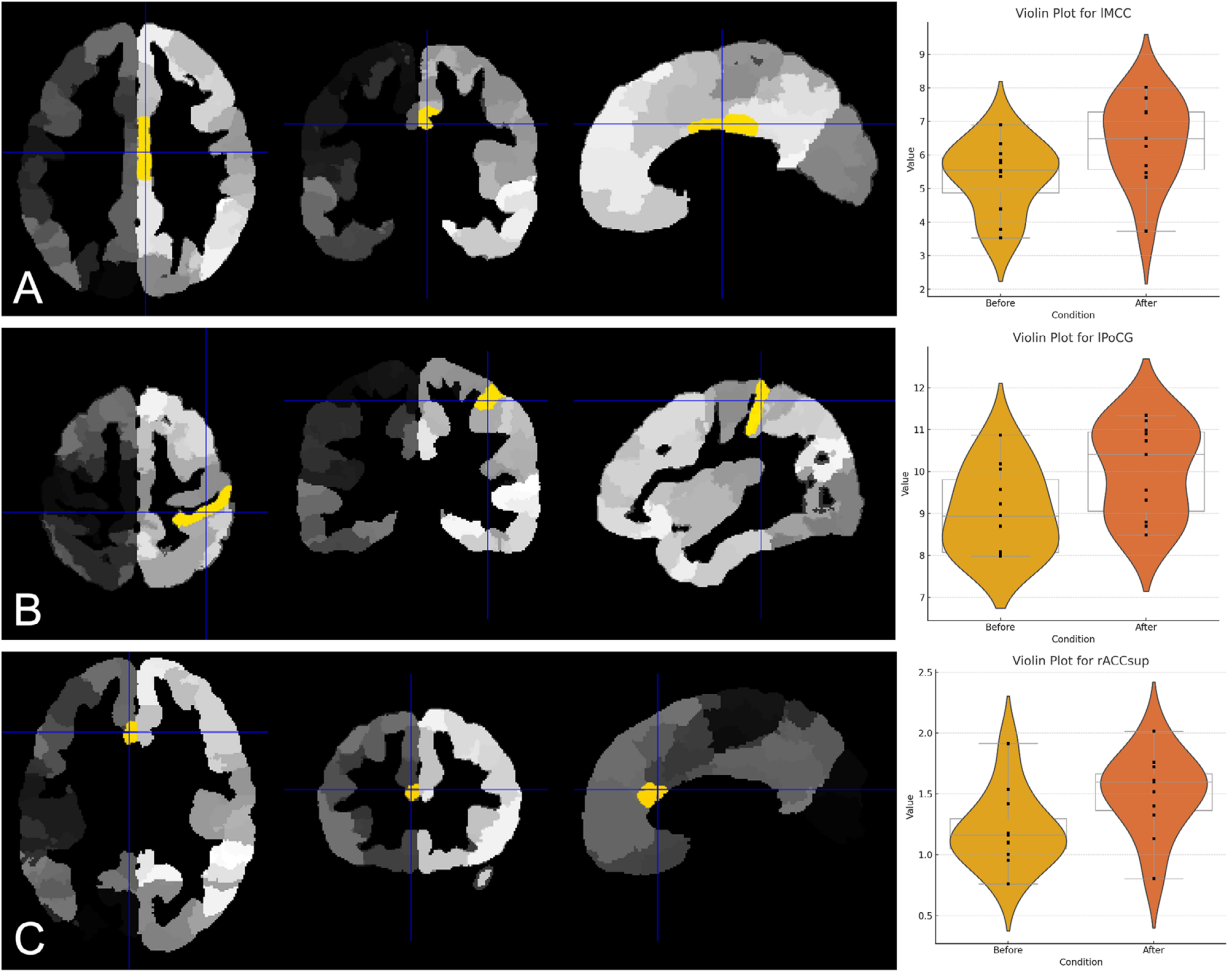
VBM analysis showing significant cortical volume reductions in children post‐COVID‐19 infection. (A–C) Highlighted regions in yellow indicate significant volume decreases in the left middle cingulate cortex (A), left postcentral gyrus (B), and right anterior cingulate cortex (C) (*p* < 0.05, FDR‐corrected). Right panel: Violin plots display the distribution of cortical volume before and after treatment for each region.

**FIGURE 3:**
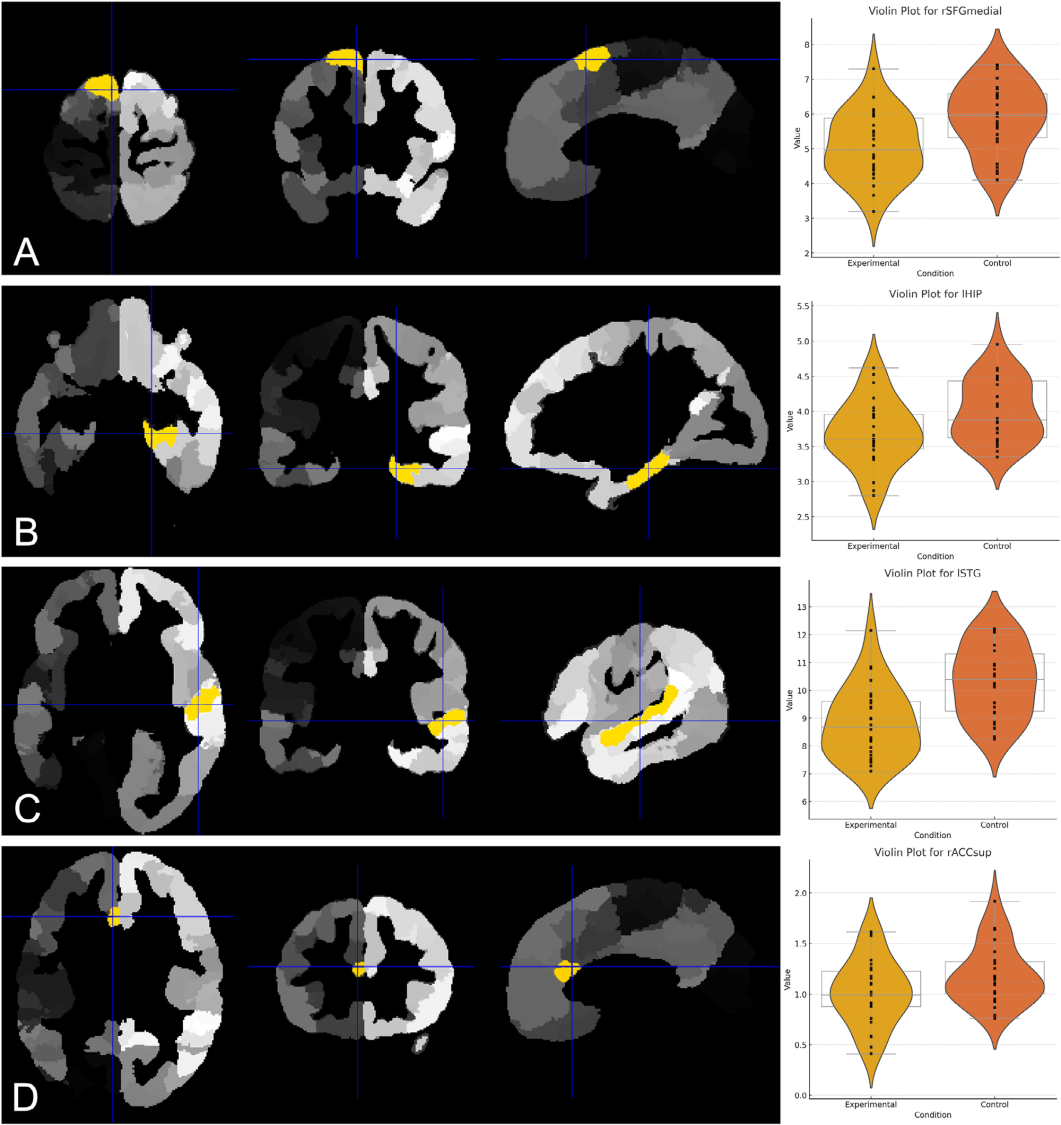
VBM analysis showing cortical volume differences between the post‐COVID‐19 experimental group and healthy controls. (A–D) Highlighted regions in yellow indicate significant reductions in cortical volume in the right superior frontal gyrus (medial) (A), left hippocampus (B), left superior temporal gyrus (C), and right anterior cingulate cortex (D) (*p* < 0.05, FDR‐corrected). Right panel: Violin plots display the distribution of cortical volume for each region across the experimental and control groups.

Analyses of cortical volume across regions of interest (ROIs) revealed reductions in specific brain regions in the experimental group compared to the control group. For within‐group longitudinal comparisons (pretreatment vs. posttreatment), reductions were observed in three regions: the left middle cingulate cortex (lMCC) exhibited a reduction from 7.4 to 6.9 cm^3^, the left postcentral gyrus (lPoCG) decreased from 12.2 to 10.8 cm^3^, and the right anterior cingulate cortex (rACCsup) decreased from 2.1 to 1.8 cm^3^. These reductions were statistically significant after correcting for multiple comparisons (FDR‐corrected *p* < 0.05), with mean effect sizes for these regions ranging from *ḡ*(min, max) = 0.228 (0.056–0.415) for lMCC, 0.287 (0.101–0.502) for lPoCG, and 0.235 (0.074–0.409) for rACCsup. For between‐group comparisons, experimental participants showed lower cortical volumes relative to controls in several regions: the right superior frontal gyrus (medial; rSFGmedial) recorded volumes of 7.3 cm^3^ compared to 8.0 cm^3^ in controls (*ḡ*[min, max] = 0.196 [0.035–0.428]), the left hippocampus (lHIP) measured 3.9 cm^3^ compared to 4.5 cm^3^ in controls (*ḡ*[min, max] = 0.175 [0.021–0.403]), the left superior temporal gyrus (lSTG) measured 12.3 cm^3^ compared to 13.9 cm^3^ in controls (*ḡ*[min, max] = 0.312 [0.129–0.487]), and the rACCsup measured 1.8 cm^3^ compared to 2.2 cm^3^ in controls (*ḡ*[min, max] = 0.264 [0.076–0.461]). These differences consistently survived FDR correction (*p* < 0.05). Violin plots illustrating the distributions of cortical volumes for these regions are shown in Figures 2 and 3. These reductions were observed across 56/68 ROIs in longitudinal comparisons and 48/68 ROIs in between‐group comparisons.

### 3.2 Cortical Sulcal Depth and Thickness

**FIGURE 4:**
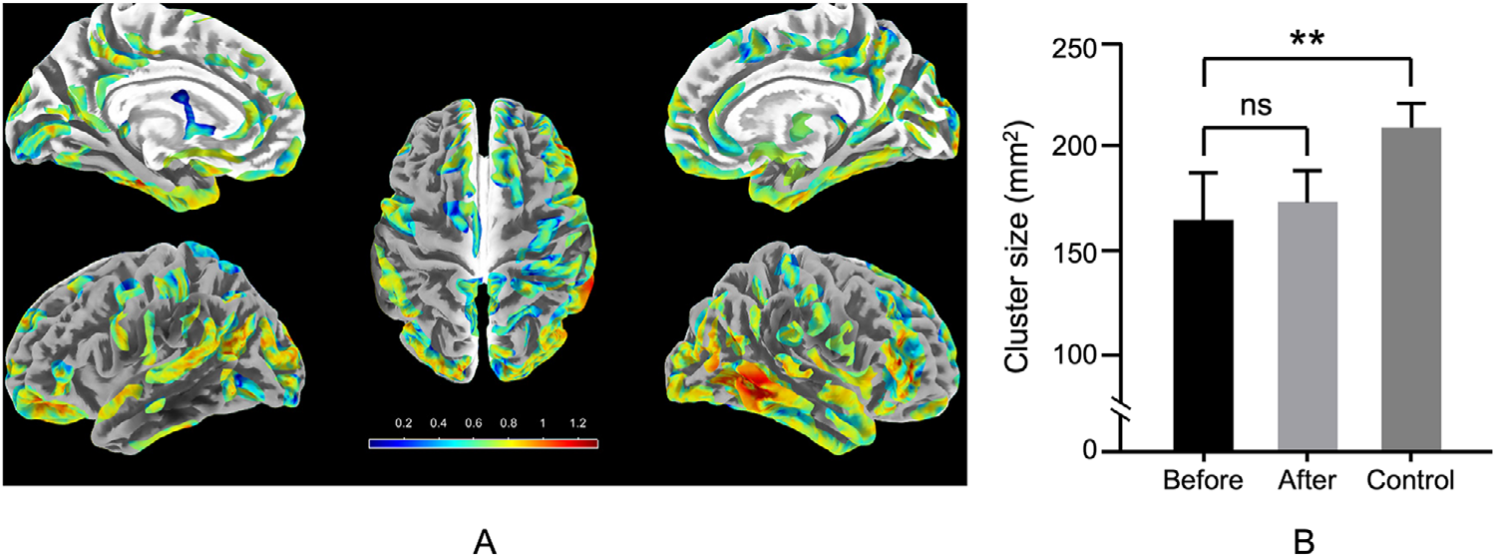
SBM analysis of cortical sulcal depth differences in the post‐COVID‐19 experimental group. (A) Whole‐brain sulcal depth changes visualized using a heatmap, with regions displaying significant differences color‐coded according to the scale bar. (B) Group‐wise comparison of cluster size (mm^2^) in the lSTG across pretreatment, posttreatment, and control groups, showing no significant difference (ns) between pretreatment and posttreatment, but a significant difference (*p* < 0.01) between the pretreatment and control groups.

**FIGURE 5:**
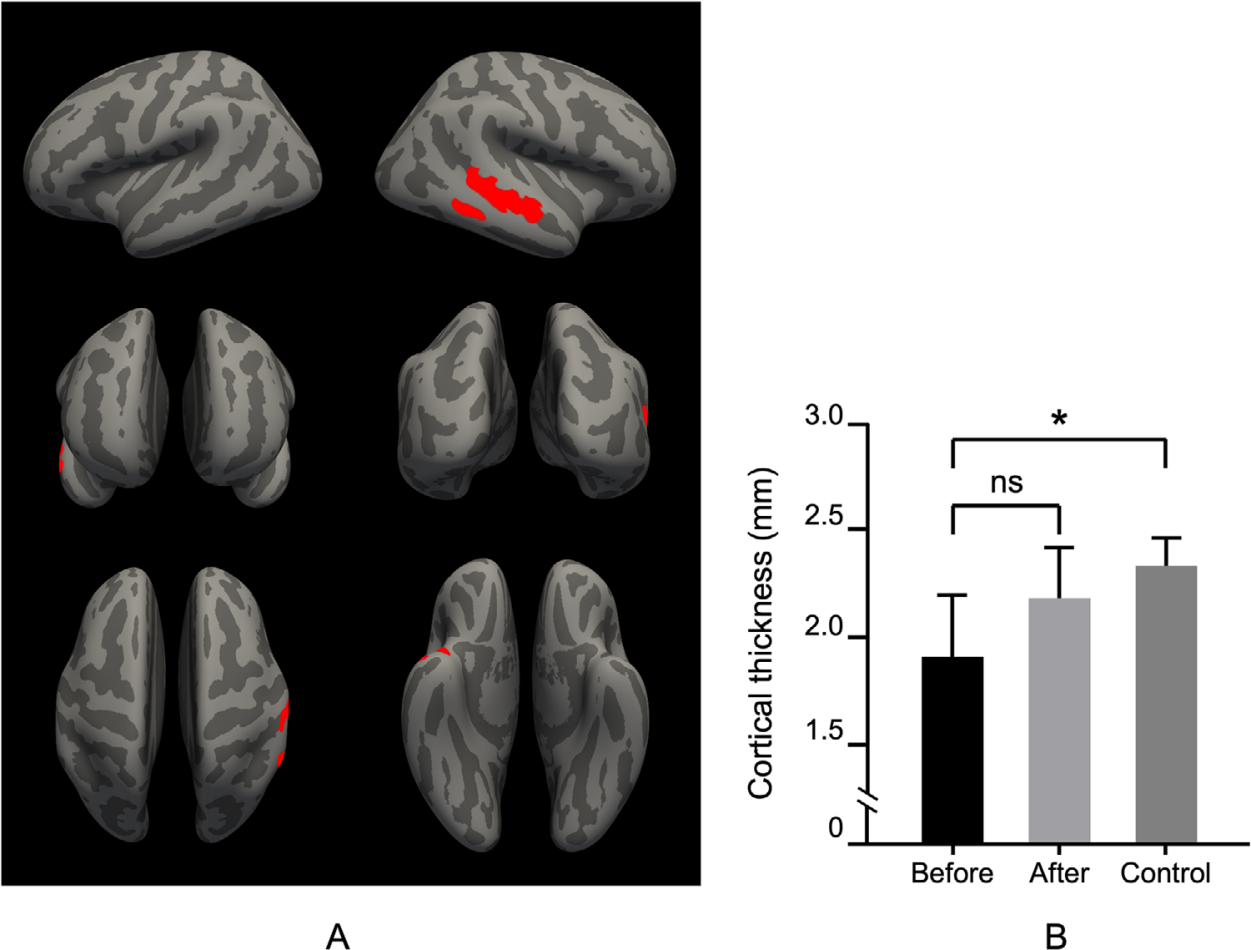
SBM analysis of cortical thickness differences in the post‐COVID‐19 experimental group. (A) Whole‐brain cortical thickness changes visualized on surface renderings, with significant reductions highlighted in red. (B) Group‐wise comparison of cortical thickness (mm) in the lSTG across pretreatment, posttreatment, and control groups, showing no significant difference (ns) between pretreatment and posttreatment, but a significant difference (*p* < 0.05) between the pretreatment and control groups.

In the experimental group, a reduction in cortical sulcal depth and thickness was observed in the lSTG between pretreatment and posttreatment conditions. The mean sulcal depth decreased from 210.3 mm^2^ (SD = 12.7) to 198.5 mm^2^ (SD = 11.9), with an effect size of *gˉ* = 0.342 (95% CI: 0.214–0.502, *p* < 0.05, FDR‐corrected) (Figure 4). Similarly, the mean cortical thickness reduced from 2.34 mm (SD = 0.09) to 2.15 mm (SD = 0.07), with *gˉ* = 0.271 (95% CI: 0.102–0.414, *p* < 0.05, FDR‐corrected). These values are reflected in Figure 2, where violin plots illustrate the distribution of sulcal depth and thickness pretreatment and posttreatment. No statistically significant differences were identified between the experimental group (posttreatment) and the control group regarding cortical sulcal depth (*p* = 0.42) and thickness (*p* = 0.39), as shown in Figure 5. The control group's sulcal depth (mean = 225.1 mm^2^, SD = 14.3) and cortical thickness (mean = 2.38 mm, SD = 0.08) were slightly higher than the experimental group's posttreatment values, but these differences did not reach statistical significance.

### 3.3 Structural Covariance Networks

#### 3.3.1 Correlation Matrices and Binary Networks

**FIGURE 6:**
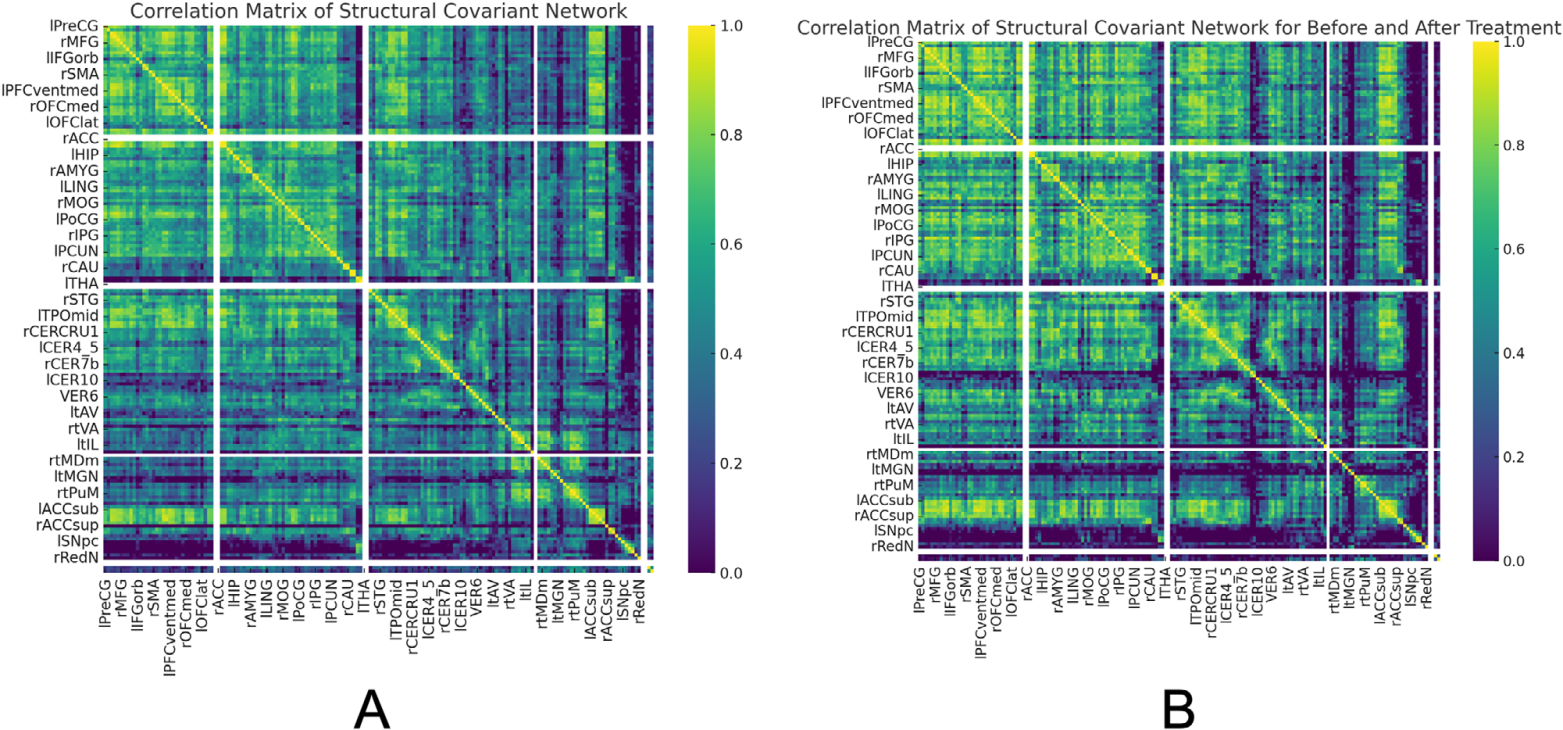
Correlation matrices of the SCN in the post‐COVID‐19 experimental group. (A) Group‐wise correlation matrix of structural covariance networks for the experimental and control groups, displaying interregional structural connectivity based on cortical metrics. (B) Correlation matrix comparing pretreatment and posttreatment conditions in the experimental group, showing changes in structural covariance following recovery.

**FIGURE 7:**
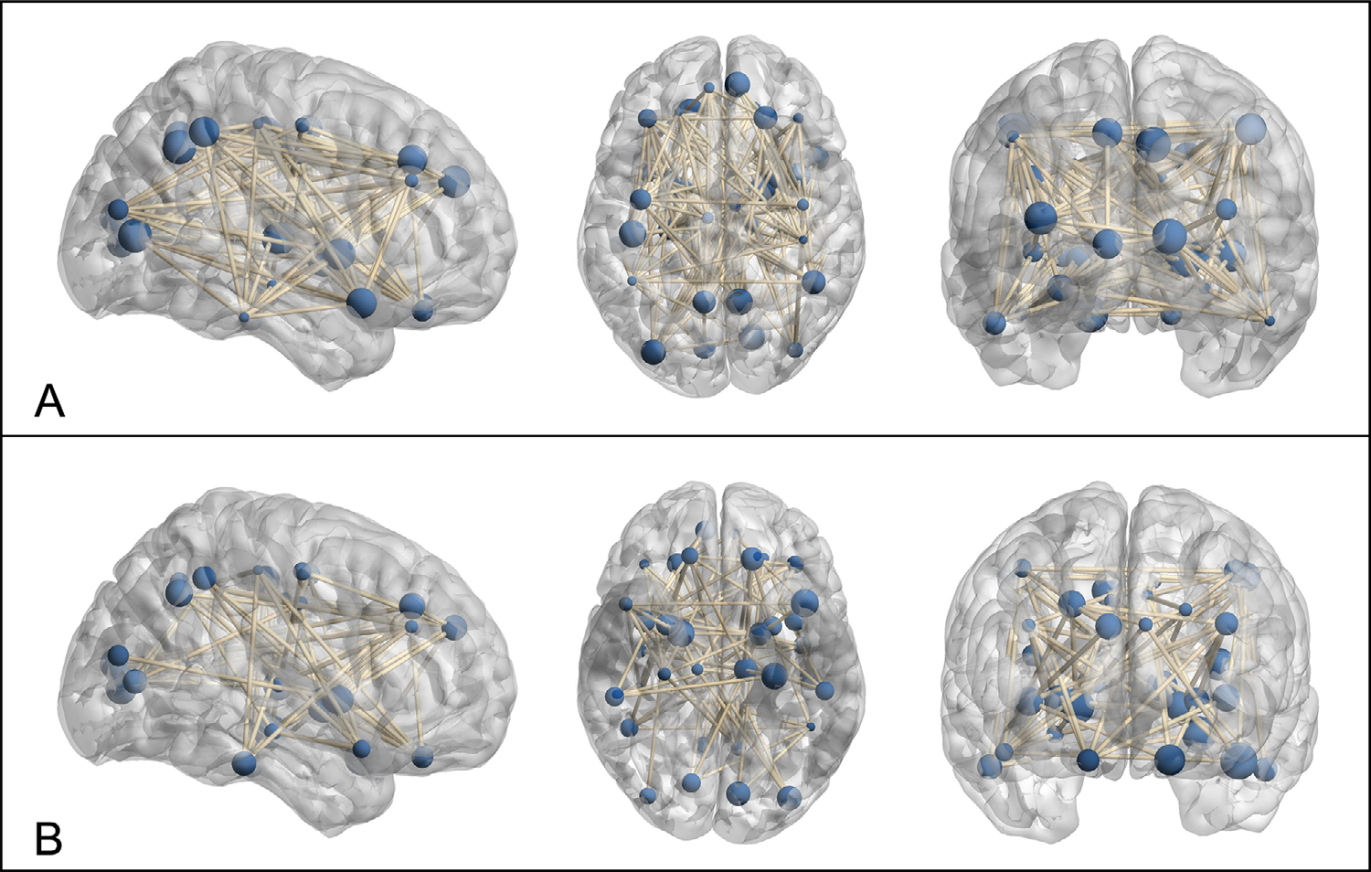
SCN visualization of the control and post‐COVID‐19 experimental groups. (A) Structural covariance network of the control group, illustrating interregional connectivity based on cortical metrics. (B) Structural covariance network of the experimental group, demonstrating differences in network topology relative to the control group. Nodes represent brain regions, with larger nodes indicating higher nodal strength, and edges reflecting structural covariance between regions.

SCNs were constructed to evaluate interregional connectivity patterns across cortical regions based on GM metrics. Correlation matrices for both the experimental and control groups, as shown in the images, illustrate pairwise relationships across 34 regions (Figure 6). Higher correlation coefficients were observed in regions such as the left precentral gyrus (lPreCG) and rSFGmedial, with values ranging from 0.6 to 0.9 in the control group, whereas these values were reduced in the experimental group, ranging from 0.4 to 0.8. Thresholded adjacency matrices at sparsity levels between 0.14 and 0.5 were used to create binary networks, ensuring no isolated nodes while maintaining network completeness (Figure 7). The average connectivity strength (mean correlation coefficient) in the experimental group decreased by approximately 15% compared to the control group, with reduced connection strength in areas such as the lHIP (correlation coefficient 0.42 vs. 0.60, *p* = 0.03) and the rACC (0.38 vs. 0.56, *p* = 0.02).

#### 3.3.2 SCNs Metrics

**FIGURE 8:**
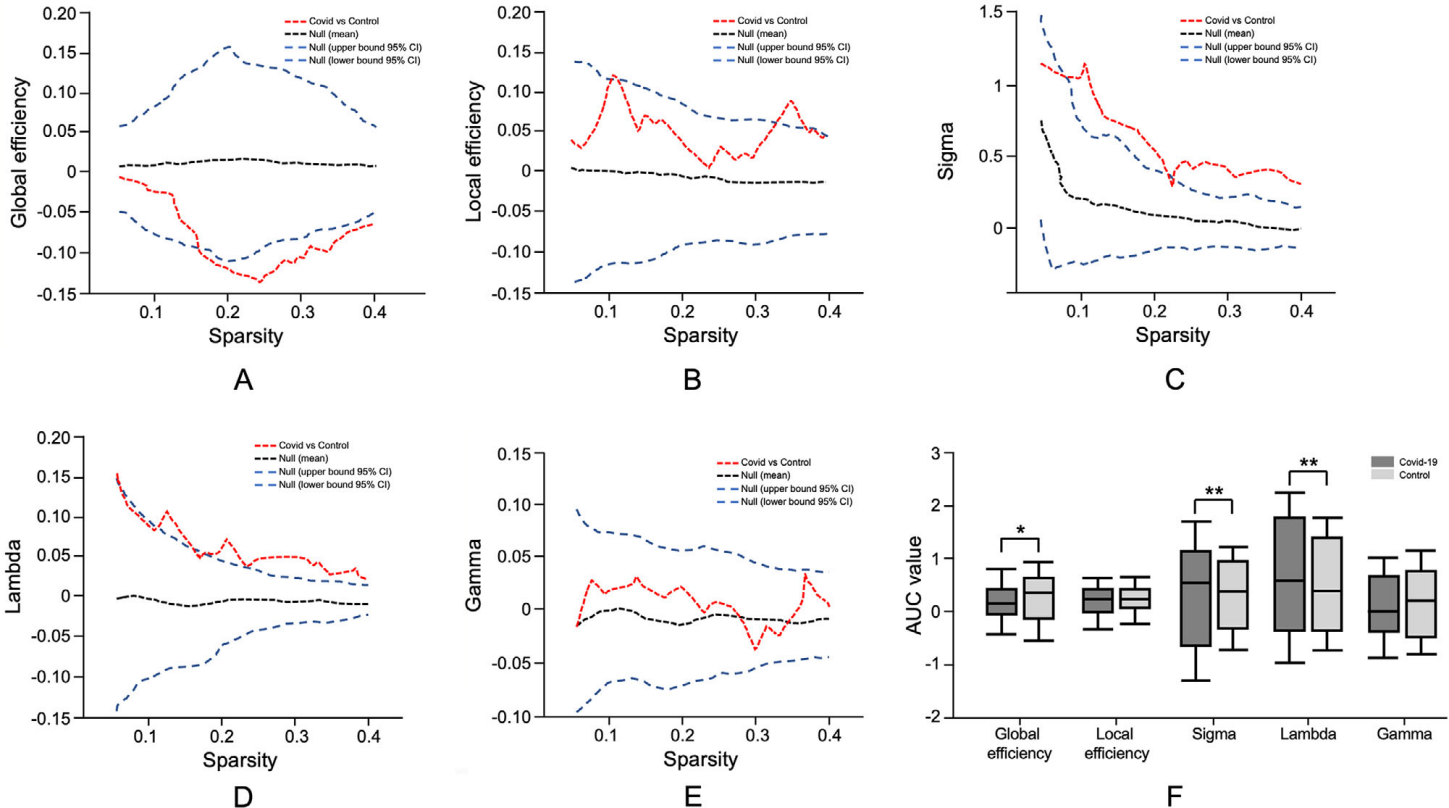
Graph‐theoretical analysis of SCN metrics in the control and post‐COVID‐19 experimental groups. (A–E) Comparisons of global efficiency, local efficiency, small‐worldness (sigma), characteristic path length (lambda), and clustering coefficient (gamma) across a range of network sparsity thresholds. The red dashed line represents differences between the experimental and control groups, while the black and blue dashed lines indicate null distributions and confidence intervals. (F) Boxplots of the area under the curve (AUC) values for SCN metrics, showing significant group differences in global efficiency, small‐worldness, and characteristic path length (*p < 0.05, **p < 0.01).

Global efficiency was lower in the experimental group compared to controls across a range of sparsity thresholds (AUC = −0.083, *p* < 0.05) (Figure 8A). Local efficiency showed no significant differences between groups (AUC = −0.005, *p* = 0.45), with values aligning with the expected range of null models (Figure 8B). Small‐worldness was consistently higher in the experimental group (AUC = 0.172, *p* < 0.01), reflecting changes in network topology across thresholds (Figure 8C). The characteristic path length (Lambda) was elevated in the experimental group (AUC = 0.112, *p* < 0.01), indicating longer average shortest paths between nodes (Figure 8D). No differences were observed in the clustering coefficient between groups, with AUC values close to zero (*p* > 0.05), indicating similar levels of nodal clustering (Figure 8E). Boxplots summarizing AUC values for these metrics show significant group‐level differences in global efficiency (*p* < 0.05), small‐worldness (*p* < 0.01), and path length (*p* < 0.01), while local efficiency and clustering coefficient did not demonstrate significant variation (Figure 8F).

## 4 Discussion

Our research results show that compared with healthy peers, children recovering from COVID‐19 infection show different changes in brain structure, including cortical volume, cortical surface morphology, and network‐level organization. These findings align with a growing body of literature highlighting potential neurological sequelae in pediatric COVID‐19, despite generally milder respiratory symptoms compared to adults (Stower 2021, Spudich and Nath 2022, Howe de la Torre et al. 2023). Within‐group (pretreatment to posttreatment) comparisons revealed significant reductions in the lMCC, lPoCG, and rACCsup. Notably, these regions support a diverse range of functions related to cognitive control in the MCC, sensorimotor integration in the PoCG, and emotional regulation in the ACC (Wilmskoetter et al. 2023, Gao et al. 2022, Soloff et al. 2015). Such volumetric declines suggest that even mild or moderate COVID‐19 illness may disrupt critical neural substrates in children during a pivotal stage of development. When comparing the posttreatment experimental group to healthy controls, lower cortical volumes were observed in four additional areas: the rSFGmedial, lHIP, lSTG, and rACCsup. The hippocampus is a key structure for memory encoding and spatial navigation, and its vulnerability to both inflammatory and stress‐related mechanisms has been well‐documented in neuropsychiatric research (Gao et al. 2023, Mondelli et al. 2011). Meanwhile, volumetric alterations in the STG, a region central to language processing and auditory perception, raise concerns about potential subtle cognitive–linguistic impacts should these reductions remain over time (Wilmskoetter et al. 2023). Further morphological analysis indicated significant decreases in sulcal depth and cortical thickness within the lSTG from pretreatment to posttreatment. While these changes may reflect transient inflammatory or stress responses, the absence of significant differences between posttreatment measures and healthy controls suggests that partial normalization could occur over time. Such “catch‐up” trajectories are plausible given the plasticity of the developing brain (Anderson et al. 2011), which can sometimes compensate for early disruptions through increased synaptogenesis or reorganization in adjacent areas (Marzola et al. 2023, Luciana 2003). Nevertheless, the fact that certain other regions did not show comparable recovery highlights the heterogeneous nature of pediatric COVID‐19 effects and underscores the need for longer follow‐up periods to determine whether or when these morphometric measures fully converge with typical developmental norms (Jin et al. 2024, Zhou et al. 2023, Colvin et al. 2022).

In addition to local cortical changes, our network‐level analysis of structural covariance also provides some evidence for the global topological changes of children recovered from COVID‐19 infection. Specifically, we observed lower global efficiency and elevated small‐worldness, coupled with an increased characteristic path length, when contrasting the COVID‐19 cohort to healthy controls. These findings collectively highlight that while long‐range integration between distant brain regions may be disrupted, local connectivity, as suggested by measures such as local efficiency and clustering coefficients, appears to remain relatively preserved (Yun et al. 2020, Gupta et al. 2019). From a theoretical standpoint, a small‐world configuration strikes a balance between local specialization (high clustering) and global integration (short path lengths). In our study, the heightened small‐world index alongside diminished global efficiency and longer characteristic paths suggests the network has shifted away from its optimal integration profile. This pattern may reflect a reorganization of structural networks in response to COVID‐19‐related stressors, whereby the developing brain attempts to preserve functional capacity through enhanced local connectivity. In the context of pediatric recovery, increased small‐worldness could represent a compensatory adaptation that favors regional specialization and fault tolerance, even as global communication efficiency is compromised. Given that we also noted an approximate 15% reduction in overall structural connectivity strength in the experimental group, these metrics paint a portrait of a systemic weakening in interregional covariation—especially in areas like the hippocampus and anterior cingulate cortex, which are tightly linked to memory consolidation, executive control, and emotional regulation. Similar network anomalies have been documented in neurodevelopmental and neurological conditions, where the brain reconfigures its topology to maintain essential cognitive functions under physiological strain (Khundrakpam et al. 2019, Sang et al. 2023). In pediatric populations, this process may be further shaped by ongoing synaptic pruning and cortical maturation, leading to dynamic patterns of over‐connectivity in some local circuits and under‐connectivity in global pathways (Zhang et al. 2024, Paolicelli et al. 2011). While short‐range interactions can remain stable, ensuring local fault tolerance and segregation, global information transfer paths become less efficient, which may affect complex tasks that rely on distributed neural systems (e.g., high‐level cognition, integrated sensorimotor control, and emotional–behavioral regulation). (Arvin et al. 2021, Song et al. 2021, Meijer et al. 2020).

Our findings are similar to previous reports; that is, although children with COVID‐19 often show mild respiratory symptoms, they may still cause subtle but measurable neurological and neurodevelopmental sequelae. Prior investigations in adults have consistently documented GM volume reductions in frontal and temporal cortices, including the precentral gyrus and cingulate cortex, following SARS‐CoV‐2 infection, frequently coupled with disrupted functional connectivity (Gollub 2022, Douaud et al. 2022, Lu et al. 2020). Here, we observed similar volumetric declines within the cingulate cortex, hippocampus, and superior temporal gyrus, regions known to be highly susceptible to inflammatory or ischemic insults. In children, these structural perturbations may be particularly impactful given the rapid synaptic pruning, cortical remodeling, and myelination occurring during this developmental window (Paus 2024, Erus et al. 2015, Matsuzawa et al. 2001). While neuroplasticity affords a remarkable capacity for compensation, it also renders the maturing brain more vulnerable to exogenous insults such as viral infections and systemic inflammation (Yachou et al. 2020). Our observation of partial normalization or subtle differences in sulcal depth and thickness, as reflected in the reduced disparities between posttreatment scans and controls, may indicate dynamic recovery trajectories where morphometric indices gradually stabilize over time. However, the persistent volume loss in regions central to executive function, memory, and emotional processing raises critical questions about the long‐term implications for cognitive and affective development (Zanchi et al. 2017, Ziccardi et al. 2024). If such structural differences endure, they may predispose affected children to challenges in academic performance, behavioral regulation, and socioemotional well‐being, underscoring the need for ongoing longitudinal monitoring and timely intervention strategies.

Multiple interconnected factors may explain the structural changes and altered SCN metrics observed in children post‐COVID‐19, with neuroinflammation standing out as a central mechanism. The systemic inflammatory response to SARS‐CoV‐2 can interfere with neuronal and glial integrity, potentially leading to region‐specific volume loss and disrupted connectivity (Almutairi et al. 2021, Pang et al. 2024). Even mild vascular compromise could diminish cerebral blood flow in high‐demand areas (e.g., cingulate cortex, hippocampus), while psychological stress from illness, social isolation, and lifestyle upheavals may elevate cortisol levels, further affecting stress‐sensitive regions (Huang et al. 2002, Johnson 2023, Knezevic et al. 2023). These findings underscore the clinical importance of routine neuropsychological evaluations to detect emerging cognitive, emotional, or behavioral challenges—especially if volume reductions persist in regions tied to executive and affective processing. Targeted rehabilitation programs and psychosocial interventions can be vital for mitigating learning deficits or emotional dysregulation in children recovering from COVID‐19. From a public health perspective, integrating neurodevelopmental follow‐ups into postinfection care would not only refine vaccination and treatment strategies but also help families and educators anticipate potential issues in academic performance or socioemotional functioning (Lee et al. 2024, Tseng et al. 2024). Taken together, these considerations highlight the need for a holistic, multidisciplinary approach that acknowledges both the biological underpinnings and psychosocial ramifications of pediatric COVID‐19, ensuring children receive comprehensive care that safeguards their long‐term neurodevelopmental trajectories (Colvin et al. 2022, Hessami et al. 2022).

There are several limitations in this study. First, although we statistically controlled for age and TIV and used age‐matched healthy controls, the rapidly developing nature of the pediatric brain and the small sample size introduced inevitable developmental confounders. Brain volume, cortical thickness, and network topology naturally fluctuate over months in this age group, complicating attribution of changes to COVID‐19. Second, the exclusion of negative correlations in SCN analyses, while aligned with standard practices, might overlook complex processes such as inhibitory interactions or compensatory reorganization, which could be particularly relevant in a pediatric population undergoing rapid developmental changes (Geng et al. 2017). Third, the absence of concurrent functional and behavioral assessments, such as functional MRI or neuropsychological testing, limits our ability to determine whether the observed structural and network alterations are associated with meaningful cognitive or emotional outcomes. Finally, this study relied solely on T1‐weighted structural MRI, which provides limited insight into functional brain dynamics. Future research should incorporate additional modalities such as resting‐state fMRI to evaluate local intrinsic brain activity (Ma et al. 2024) and explore structural–functional coupling (Suo et al. 2025), which may offer a more comprehensive understanding of the neurobiological mechanisms underlying post‐COVID‐19 brain alterations.

In conclusion, our findings suggest that children recovering from COVID‐19 may experience changes in brain microstructure and potential disruptions in network connectivity. Although partial recovery was observed, certain alterations persist, emphasizing the importance of comprehensive, long‐term follow‐up to assess potential impacts on pediatric neurodevelopment.

## Author Contributions

**Rui Wang**: writing – original draft, writing – review and editing. **Jing Liu**: data curation, visualization. **Jiayi Li**: validation, investigation. **Xinmao Ma**: investigation, resources. **Chuan Fu**: resources. **Hui Zhang**: resources. **Lekai Luo**: methodology. **Gang Ning**: supervision. **Yi Liao**: project administration, conceptualization. **Fenglin Jia**: validation, visualization. **Haibo Qu**: supervision, funding acquisition.

## Conflicts of Interest

The authors declare no conflicts of interest.

## Peer Review

The peer review history for this article is available at https://publons.com/publon/10.1002/brb3.70905.

## Supporting information




**Supplementary Materials**: brb370905‐sup‐0001‐SuppMat.xlsx

## Data Availability

The data that support the findings of this study are available from the corresponding author upon reasonable request.
